# Real-time multi-class threat detection and adaptive deception in Kubernetes environments

**DOI:** 10.1038/s41598-025-91606-8

**Published:** 2025-03-15

**Authors:** Abdelrahman Aly, Ahmed M. Hamad, Mirvat Al-Qutt, Mahmoud Fayez

**Affiliations:** Faculty of Computer and Information Science, Ain Shams University, Cairo, Egypt

**Keywords:** Kubernetes security, Multi-class detection, Cyber deception, KServe, Real-time threat detection, Adaptive security, Network traffic analysis, Cloud-native infrastructure, Computer science, Information technology

## Abstract

Kubernetes has emerged as the backbone of modern cloud-native environments, enabling efficient orchestration of containerized applications. However, its dynamic nature exposes it to sophisticated cyber threats, including privilege escalation, reconnaissance, and denial-of-service attacks. This paper presents a novel framework that combines real-time multi-class threat detection with adaptive deception to enhance Kubernetes security. The framework integrates KServe for scalable machine learning-based threat classification, CICFlowMeter for feature extraction, and KubeDeceive for dynamic deployment of decoys, all governed by the MAPE-K loop for continuous adaptation. Evaluations demonstrate high detection accuracy (up to 91%), efficient resource utilization, and effective attacker engagement, with decoy success rates reaching 93%. The results underscore the framework’s ability to proactively mitigate threats, maintain system resilience, and provide actionable intelligence. This unified approach represents a scalable and adaptable defense mechanism for Kubernetes environments, catering to the needs of dynamic and resource-intensive cloud infrastructures.

## Introduction

Kubernetes^1^ has become the backbone of modern cloud-native infrastructure, enabling efficient orchestration of containerized applications at scale. However, its dynamic and complex nature introduces significant security challenges, making it a prime target for advanced cyber threats. These include access exploits such as privilege escalation and improper authentication vulnerabilities, application-layer attacks like server-side request forgery (SSRF)^2^ and path traversal^3^, container-specific threats such as container escapes, and disruptive activities including denial-of-service (DoS) and reconnaissance attacks. These threats jeopardize critical services, potentially exposing sensitive data, compromising interconnected systems, or causing widespread service disruptions.

Existing security solutions, such as traditional signature-based and anomaly detection systems, struggle to address these threats in dynamic Kubernetes environments. Signature-based systems are unable to detect novel or zero-day attacks, while anomaly detection systems suffer from high false positive rates, overwhelming security teams with irrelevant alerts. The limitations of these approaches, coupled with the distributed and fast-paced nature of Kubernetes workloads, demand a more adaptive and integrated defense mechanism.

This paper presents a unified framework that combines real-time multi-class threat detection with adaptive cyber deception to address these challenges. At a high level, the framework leverages machine learning to classify network traffic, deploys tailored decoy resources to mislead attackers, and continuously adapts its strategies using the MAPE-K^4^ loop (Monitor, Analyze, Plan, Execute, and Knowledge). This integration enables precise threat detection, proactive engagement of adversaries, and real-time incident response, reducing false positives and enhancing the overall security of Kubernetes environments.

By bridging the gap between detection and deception, this approach empowers Security Operations Centers (SOCs)^5^ to counter evolving attack strategies effectively. The framework has been rigorously evaluated using comprehensive metrics, including detection accuracy, response latency, false positive rates, and resource utilization. These evaluations highlight the framework’s ability to provide reliable, real-time threat detection and adaptive defense, ensuring strong protection for Kubernetes clusters in dynamic and high-traffic environments.

This paper is organized as follows: Section 1 introduces the framework and its motivation. Section 2 reviews related works. Section 3 details the framework’s design and components. Section 4 covers its implementation and operational methodology. Section 5 presents the experimental evaluation, and Section 6 concludes with key findings and future directions.

## Related work

As Kubernetes becomes a critical component of modern cloud-native infrastructure, securing these environments has attracted significant research attention. Existing studies have explored various approaches, including machine learning-based anomaly detection, signature verification techniques, and adaptive deception frameworks. While these works offer valuable contributions to specific aspects of cybersecurity, they often fail to address the unique challenges posed by Kubernetes, such as its dynamic workloads, distributed architecture, and real-time operational demands. This section reviews key contributions from the literature, highlighting their methodologies, strengths, and limitations, and establishes the need for a unified framework that integrates multi-class threat detection and adaptive deception to address these gaps effectively.

Johnson et al. (2024)^6^ propose a deception-based framework titled “ADAPT: Adaptive Camouflage-Based Deception Orchestration for Trapping Advanced Persistent Threats,” designed to counter APTs^7^ by leveraging adaptive camouflage techniques. The framework orchestrates behavioral honeypots that simulate realistic system responses and dynamically modify attack paths based on detected attacker behavior. While this approach is effective at engaging attackers and gathering intelligence on their tactics, techniques, and procedures (TTPs)^8^, its focus is confined to APT-specific threats. Furthermore, the system’s reliance on predefined deception orchestration paths diminishes its adaptability in highly dynamic environments like Kubernetes, where workloads and threat vectors evolve rapidly. The lack of integration with broader multi-class detection mechanisms further limits its applicability to detecting diverse threat types, such as reconnaissance or denial-of-service (DoS) attacks. These limitations underscore the need for a system that seamlessly integrates adaptive deception with robust, real-time detection capabilities.

Building on machine learning-based techniques for anomaly detection, Sharma et al. (2022)^9^ introduce “A Real-Time Adaptive Network Intrusion Detection for Streaming Data: A Hybrid Approach,” which combines K-Nearest Neighbors (KNN) and Naive Bayes classifiers to detect anomalies in streaming network traffic. The model utilizes feature selection techniques to focus on 16 critical attributes, achieving high detection accuracy while minimizing computational overhead. Evaluated on NSL-KDD and KDD 1999 datasets, the system demonstrates strong performance for both known and unknown threats. However, its dependence on static datasets and predefined feature sets restricts its adaptability to the dynamic and high-speed traffic typically observed in Kubernetes clusters. Furthermore, the model lacks mechanisms to incorporate evolving threat intelligence or engage attackers post-detection, making it less effective for real-time scenarios. These gaps highlight the necessity of a Kubernetes-specific system capable of adapting to evolving attack patterns while integrating detection with proactive defenses like deception.

Kudo et al. (2022)^10^ propose “Application Integrity Protection on Kubernetes Clusters Based on Manifest Signature Verification,” which enhances Kubernetes application security by validating resource manifests during the admission process. This system ensures that only authorized configurations are deployed, achieving an integrity protection rate of 98.22%. While effective at blocking unauthorized deployments, the approach is primarily reactive, addressing deployment-time security without covering runtime detection or mitigation of ongoing threats. Additionally, the signature verification process introduces performance overhead, which can become a bottleneck in large-scale Kubernetes clusters with high request volumes. These limitations indicate the need for a proactive solution that integrates real-time threat detection with deception techniques to protect Kubernetes environments during and after deployment.

**Table 1:** Comprehensive comparison of proposed framework with existing solutions.

Study	Detection Approach	Detection Accuracy	Response Time	Real-Time Detection	Deception Integration	Kubernetes-Specific Dataset	Adaptability	Proactive Engagement
A Real-Time Adaptive Network Intrusion Detection for Streaming Data (2022)	Hybrid (KNN + Naive Bayes)	85% (NSL-KDD)	High	Supported	Not Integrated	Not Included	Low (Static Models)	None
Machine Learning Algorithms for Raw and Unbalanced Intrusion Detection Data in a Multi-Class Classification Problem (2023)	Decision Trees, Random Forest, CNN	96% (CIC-IDS2017)	Moderate	Supported	Not Integrated	Not Included	Low (Offline Models)	None
ADAPT: Adaptive Camouflage-Based Deception Orchestration for Trapping Advanced Persistent Threats (2024)	None	N/A	N/A	Not Supported	Integrated	Not Included	Low (Static Templates)	High
AI-Powered Anomaly Detection for Kubernetes Security: A Systematic Approach to Identifying Threats (2024)	AI-Based Anomaly Detection	92%	Moderate	Supported	Not Integrated	Included	Low (High False Positives)	None
Application Integrity Protection on Kubernetes Cluster Based on Manifest Signature Verification (2022)	Signature Verification	98.22%	High	Not Supported	Not Integrated	Not Included	None	None
Integrating Signature Apriori-Based Network Intrusion Detection System (NIDS) in Cloud Computing (2012)	Signature-Based Detection	88% (Simulated Traffic)	High	Not Supported	Not Integrated	Not Included	None	None
**Proposed Framework**	**ML-Based Detection + Deception**	**91% (Real-Time)**	**Low**	**Supported**	**Integrated**	**Included**	**High (Dynamic)**	**High**

Gupta et al. (2023)^11^ address the challenge of imbalanced datasets in intrusion detection through their study “Machine Learning Algorithms for Raw and Unbalanced Intrusion Detection Data in a Multi-Class Classification Problem.” The study evaluates machine learning models, including Decision Trees, Random Forest, and Convolutional Neural Networks (CNNs), using CIC-IDS2017 and CSE-CIC-IDS2018 datasets. Their results highlight high classification accuracy, with Decision Trees achieving a macro F1-score of 0.96878 for a 28-class task. Additionally, the integration of explainable AI (XAI) techniques enhances the interpretability of the models by identifying the most influential features. Despite these advances, the study relies exclusively on offline datasets and static environments, limiting its applicability to containerized environments like Kubernetes, where real-time classification and adaptability to evolving threats are critical. These shortcomings further underscore the need for an integrated solution that combines real-time detection with active deception to address modern cloud-native security challenges.

Bhardwaj et al. (2024)^12^ present “AI-Powered Anomaly Detection for Kubernetes Security: A Systematic Approach to Identifying Threats,” which leverages advanced machine learning algorithms to detect anomalous behaviors in Kubernetes environments. By analyzing real-time telemetry data from clusters, the system achieves a detection rate of 92% for both known and unknown threats. The study focuses on unsupervised learning models, which can adapt to various patterns of behavior in Kubernetes workloads. However, these models suffer from a high rate of false positives, primarily due to the inherent variability in Kubernetes operations. This limitation can overwhelm security teams with alerts, reducing the system’s overall efficiency. Furthermore, the study lacks integration with defensive strategies, such as deception, to proactively respond to detected anomalies. These shortcomings emphasize the need for an approach that not only identifies threats with precision but also engages attackers to mitigate risks dynamically.

Modi et al. (2021)^13^ introduce a signature-based intrusion detection framework titled “Integrating Signature Apriori-Based Network Intrusion Detection System (NIDS) in Cloud Computing,” which enhances traditional NIDS by integrating it with a signature Apriori algorithm. This approach enables the system to generate new detection rules dynamically from captured network packets, improving its ability to identify derivative attacks. The study demonstrates robust performance against known attack vectors by leveraging Snort for rule generation and classification. However, its dependence on pre-existing signatures makes it ineffective against zero-day threats and novel attack patterns, a critical limitation in the dynamic and evolving threat landscape of Kubernetes environments. Additionally, the system lacks real-time adaptability and proactive engagement mechanisms, reducing its applicability to highly automated Kubernetes clusters.

The reviewed studies collectively demonstrate significant advancements in threat detection and security strategies for both cloud and Kubernetes environments. Approaches such as machine learning-based anomaly detection (e.g., Sharma et al. and Bhardwaj et al.), signature verification mechanisms (e.g., Kudo et al. and Modi et al.), and adaptive deception frameworks (e.g., Johnson et al.) have addressed specific aspects of cybersecurity. However, these methods reveal critical limitations when applied to Kubernetes clusters. Specifically, the reliance on static datasets, predefined feature sets, and reactive mechanisms hinders their adaptability to the dynamic, high-speed, and evolving traffic patterns of Kubernetes. Additionally, many of these systems lack integration with proactive defensive measures, such as deception, which are essential for engaging attackers post-detection and mitigating their impact in real time.

In contrast, the proposed framework combines real-time multi-class threat detection, adaptive deception strategies, and dynamic adaptability through the MAPE-K loop to address these limitations comprehensively. By achieving detection accuracy of up to 91% and seamlessly integrating Kubernetes-specific defenses, our framework offers a scalable and practical solution for modern cloud-native environments. The comparative table below summarizes the gaps in the existing works and highlights the distinct advantages of our framework.

As summarized in Table 1, existing solutions demonstrate strengths in specific areas but often fail to deliver a unified, real-time approach to threat detection and defense. The proposed framework bridges these gaps by integrating machine learning-based classification with adaptive deception tailored to Kubernetes environments. With its ability to support real-time detection, proactively engage attackers, and dynamically adapt to evolving threats, this framework provides a comprehensive and effective solution, setting it apart from prior works.

## Framework design and components

In this chapter, we introduce the integrated framework that combines multi-class attack detection with cyber deception to enhance the security of Kubernetes environments. The framework builds on the foundational work from two previous research papers:

- **“**Multi-Class Threat Detection Using Neural Network and Machine Learning Approaches in Kubernetes Environments”^14^, which presented a novel multi-class detection model capable of identifying and classifying a wide range of security threats within Kubernetes clusters using neural networks and feature extraction techniques like Principal Component Analysis (PCA)^15^ and Autoencoders^16^.
- **“**KubeDeceive: Unveiling Deceptive Approaches to Protect Kubernetes Clusters”^17^, which proposed a deception-based security framework designed to intercept and redirect malicious actions targeting Kubernetes clusters. KubeDeceive deploys decoy resources in real time to mislead attackers, extend their engagement, and gather intelligence on their behaviors.

Building upon these previous works, this chapter details how we integrate both detection and deception mechanisms within Kubernetes to provide real-time protection across multiple phases of an attack. This integration is aimed at improving SOC Layer 2 efficiency, increasing deception accuracy, and providing a proactive defense layer against known vulnerabilities (CVEs)^18^ in Kubernetes.

### Framework components and interaction

**Fig. 1:**
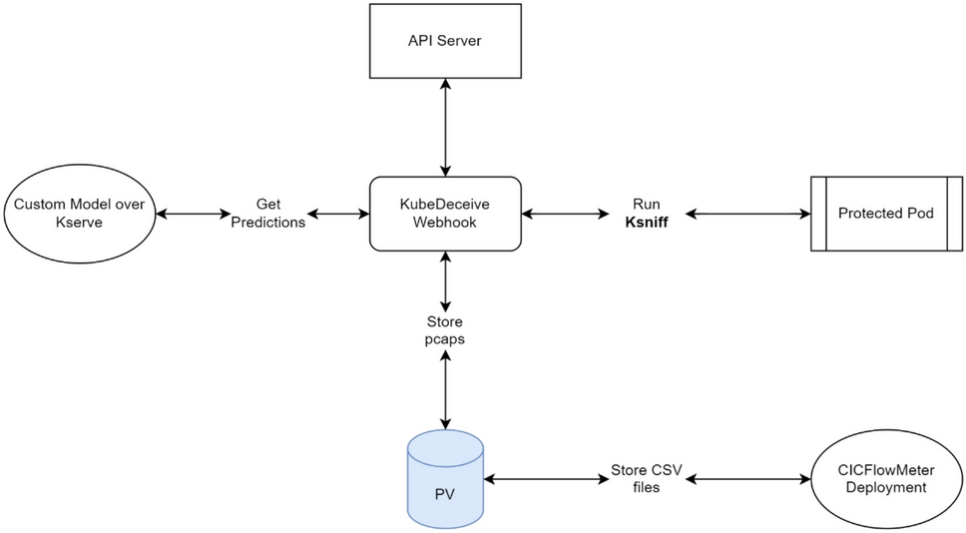
Workflow of the KubeDeceive enhanced framework with multi-class detection model.

The proposed framework integrates key components to create a seamless and dynamic defense system for Kubernetes environments. Each component plays a vital role, interacting cohesively to address real-time threats and adapt to evolving attack patterns. Figure 1 illustrates the collaborative roles of these components in providing comprehensive security. Source code is available at^19^.

- **KSniff**^20^ : This network packet capture tool intercepts live traffic from Kubernetes pods, collecting it directly from cluster interfaces. The captured traffic is stored in Persistent Volumes (PVs)^21^ for reliable and scalable access during subsequent analysis.
- **Persistent Volumes (PVs)**: PVs provide consistent and scalable storage for captured .pcap files and processed NetFlow data. They enable the framework to handle high data volumes without performance degradation, ensuring smooth integration with downstream components like CICFlowMeter^22^.
- **CICFlowMeter:** This component processes raw traffic data from .pcap files stored in PVs, transforming it into structured NetFlow features such as packet lengths, flow durations, and protocol types. These features are essential for accurate traffic classification.
- **KServe**^23^: KServe hosts the machine learning models responsible for classifying traffic in real-time. It processes features generated by CICFlowMeter to determine whether traffic is benign or malicious. With Kubernetes-native scalability, KServe dynamically adjusts resource allocation to maintain high performance under varying workloads.
- **KubeDeceive:** Serving as the proactive defense layer, KubeDeceive dynamically deploys decoys that mimic vulnerable services or sensitive resources. These decoys engage attackers, divert malicious actions away from real assets, and gather intelligence for refining defense strategies. Integration with the detection pipeline ensures that decoys are deployed based on real-time threat analysis.
- **MAPE-K Loop:** The Monitor, Analyze, Plan, Execute, and Knowledge (MAPE-K) loop drives the framework’s adaptability. By continuously monitoring traffic patterns, attacker behaviors, and system performance, it dynamically adjusts detection thresholds, deploys additional decoys, and scales resources. This feedback-driven mechanism ensures the framework remains responsive to new and evolving threats.

The integrated design of the framework allows it to seamlessly combine detection, deception, and adaptive response mechanisms, setting it apart from traditional static detection systems. By unifying these components, the framework addresses the limitations of existing solutions, including their reliance on static datasets, lack of proactive engagement, and limited scalability. This integration ensures that Kubernetes clusters remain resilient against a wide range of threats, offering a robust and scalable defense mechanism tailored to modern cloud-native environments. Having outlined the core components of the framework, we now delve into the detection pipeline, which serves as the first line of defense.

### Multi-class attack detection

The detection component of the framework enables real-time classification of diverse threats in Kubernetes environments. By leveraging Principal Component Analysis (PCA) and a Naive Bayes classifier, the system achieves high accuracy and low latency. Structured NetFlow features derived from raw traffic data allow precise differentiation between benign and malicious activities, including reconnaissance, privilege escalation, and CVE-specific exploits. The following subsections detail the detection model’s design, testing, and deployment, highlighting its critical role in the framework’s threat response capabilities.

#### Detection model development and testing

The detection model is built on a diverse and comprehensive dataset specifically designed for Kubernetes environments. This dataset captures the complexity and variety of real-world traffic patterns, ensuring the model’s effectiveness across multiple attack scenarios and benign activities. Key features of the dataset include:

- Real-World CVEs: The dataset captures attack scenarios from vulnerabilities such as:
  - CVE-2020-13379: Grafana SSRF vulnerability.
  - CVE-2019-5736: RunC command injection.
  - CVE-2019-20933: InfluxDB improper authentication.
  - CVE-2021-25741: Kubernetes kubelet race condition.
  - CVE-2021-30465: Runc symlink attack.
- Attack Scenarios: The dataset includes 10 crafted scenarios, each reflecting distinct attack types. For instance, CVE-2020-13379 exploited the Grafana service through server-side request forgery, while CVE-2019-5736 targeted the RunC binary for command injection.
- Benign Traffic: Traffic generated using OWASP ZAP’s Ajax Spider ensures realistic baseline patterns, helping differentiate normal operations from malicious activities.
- Environment Setup: Data collection occurred on a Kubernetes cluster with vulnerable components (e.g., Kubernetes v1.20.0, containerd v1.6.0), capturing internal and external traffic through tcpdump on the CNI plugin interface.
- Feature Engineering: Raw .pcap data was transformed into structured features, such as packet lengths, flow durations, protocol types, and flags, which are crucial for identifying anomalies and attack patterns.

This diverse and targeted dataset ensures the model is robust and adaptable to real-world Kubernetes workloads, allowing it to accurately distinguish between benign and malicious traffic.

**Table 2:** Performance of different model configurations for multi-class detection.

Model Configuration	Precision	Recall	F1 Score
Naive Bayes with PCA	0.94	0.91	0.93
Naive Bayes with Autoencoders	0.94	0.89	0.92
Naive Bayes with PCA + Autoencoders	0.94	0.97	0.95

The Naive Bayes with PCA and Autoencoders configuration was selected as the most effective, optimizing the classification pipeline for precision and scalability. This robust design underpins the framework’s capability to deliver real-time insights into network threats while supporting adaptive deception strategies.

The detection model underwent rigorous local testing during its development phase to ensure robustness and scalability. MLServer was used as a lightweight hosting and testing environment for pre-trained components, including PCA, autoencoders, and the Naive Bayes classifier. Serialized .pkl files were employed to streamline integration into the testing pipeline.

Key aspects of the testing process included:

- Pipeline Validation: Ensuring that input data was correctly preprocessed, transformed, and classified within the integrated model pipeline.
- Data Robustness: Testing the model against diverse netflows, including benign traffic and various attack scenarios, to confirm its ability to distinguish between normal and malicious activities.

As shown in Table 2, the model achieved an F1 score of 0.95 and a detection accuracy of up to 91% when combining PCA, autoencoders, and the Naive Bayes classifier. These results validate the model’s effectiveness in maintaining high precision and scalability in Kubernetes environments. Metrics such as false positive rates and latency were also monitored, ensuring the model met the requirements for real-time operations.

#### Deployment with KServe

The deployment of the multi-class detection model on KServe was a critical step in integrating the framework’s real-time threat detection and adaptive capabilities within Kubernetes environments. The deployment relied on a custom server environment built to handle the specific needs of Kubernetes-based operations. This environment comprises the CustomModel, which is the core model hosted by KServe, and the CustomModelServer, which interfaces with incoming HTTP requests to provide robust and scalable support for real-time traffic classification.

The CustomModel is responsible for loading pre-trained components-such as PCA, Autoencoders, and the Naive Bayes classifier-from .pkl files. It preprocesses incoming data, applying feature scaling and encoding, and performs real-time classification of network traffic. Acting as the backbone of the detection process, the CustomModel ensures efficient and accurate analysis of traffic patterns. Meanwhile, the CustomModelServer serves as the intermediary between the model and external requests. It manages multiple models in memory to optimize resource utilization and handles HTTP POST requests via a RESTful API, ensuring seamless interaction with other components.

**Fig. 2:**
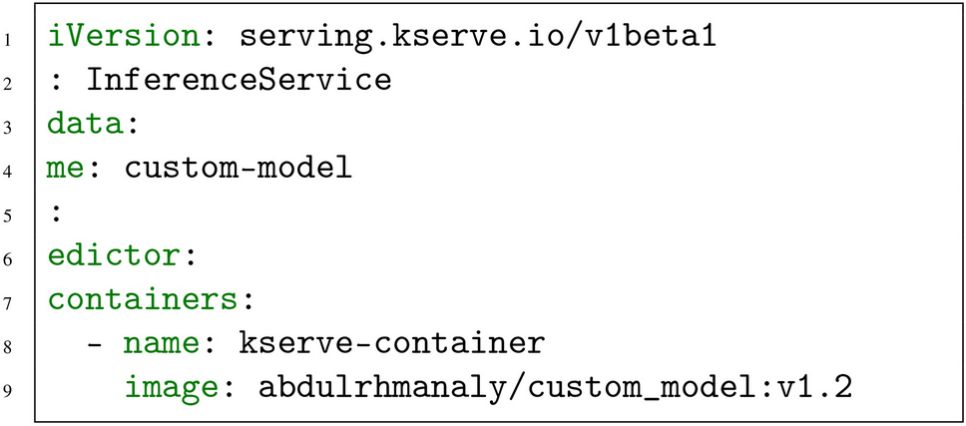
KServe InferenceService YAML configuration.

To deploy the CustomModel on KServe, the following steps were executed. First, the model and server, along with their dependencies and pre-trained files, were containerized using Docker. This containerization ensured consistency and portability across environments. Next, a YAML configuration file was created to define deployment parameters, including the container image location, resource requests, and model inference endpoints. The YAML configuration is shown below in Fig. 2.

After preparing the Docker image and configuration, the container was pushed to a container registry and deployed to the Kubernetes cluster using KServe. KServe’s InferenceService managed the deployment, providing endpoints for real-time traffic classification and enabling dynamic scaling based on workload demands.

Validation of the deployed model involved rigorous testing with both benign and malicious traffic scenarios. Metrics such as prediction accuracy, response latency, and resource utilization were carefully monitored to ensure the deployment met performance expectations. While detection is critical, the framework also incorporates an adaptive deception mechanism to complement it, as detailed in the following section.

### Adaptive deception mechanism with KubeDeceives

#### Dynamic adaptation and role of KubeDeceive

KubeDeceive enhances Kubernetes security by dynamically deploying decoys that simulate vulnerable resources, such as fake APIs, credentials, or misconfigured services. These decoys mislead attackers, diverting them from legitimate resources while gathering valuable intelligence about their actions. By integrating closely with the detection pipeline, KubeDeceive ensures real-time protection against evolving threats while supporting informed decision-making.

The adaptability of KubeDeceive is inspired by the principles of the MAPE-K loop. Although not technically implemented in this framework, the conceptual use of MAPE-K allows for dynamic adjustments based on continuous feedback from the system. Observed traffic patterns and attacker behaviors guide the deployment of tailored decoys, ensuring they remain effective in engaging adversaries. For example, during reconnaissance activities, the system can increase the density of decoys to simulate more targets, while specific attacks targeting services like Grafana or InfluxDB trigger decoys that mimic those services.

**Fig. 3:**
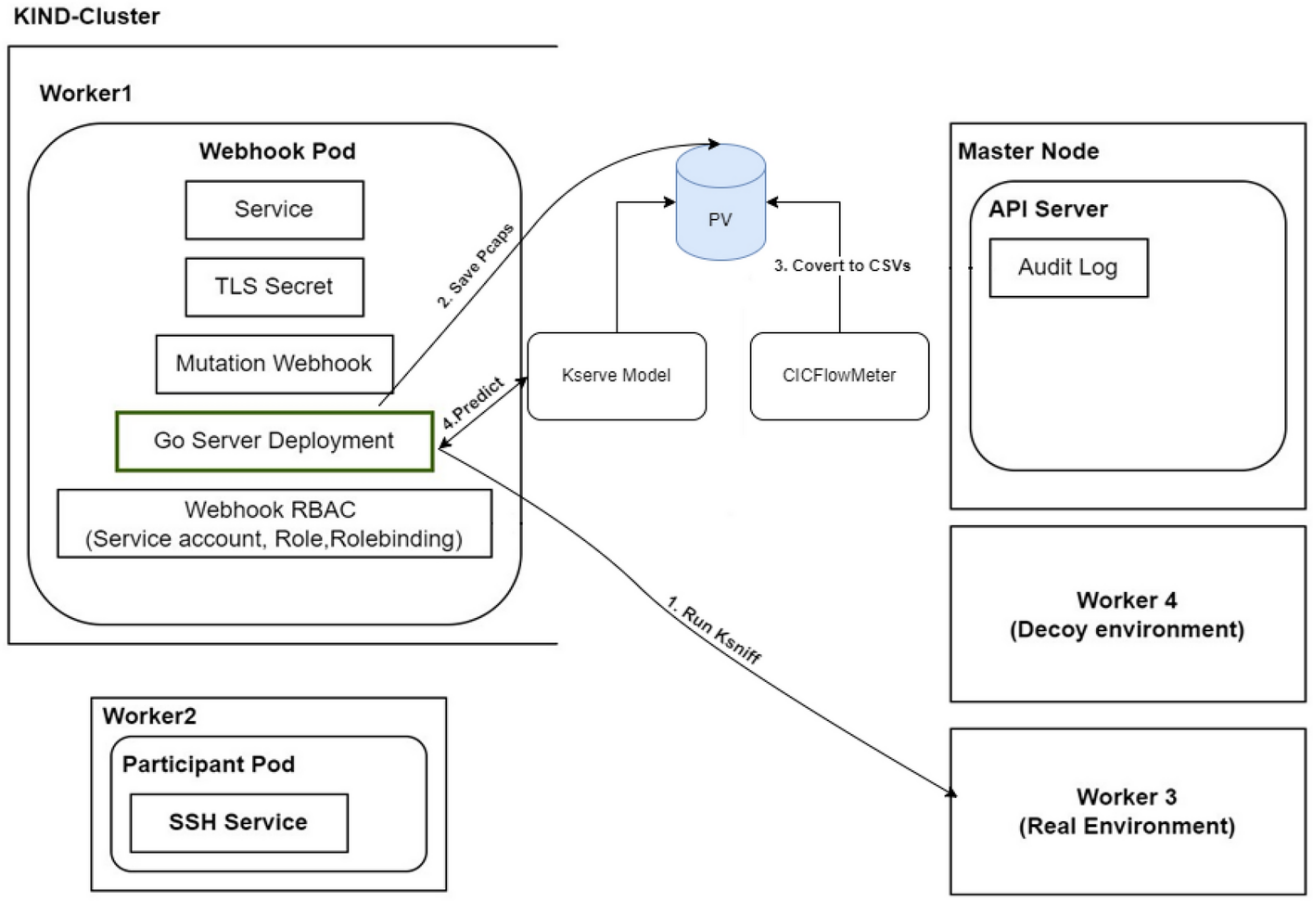
KubeDeceive with model integration. This figure depicts the architecture of a KIND-Cluster incorporating KubeDeceive.

Figure 3 illustrates how KubeDeceive integrates into the framework. Network traffic is first captured by KSniff and processed by CICFlowMeter to extract relevant features. These features are then classified by a multi-class detection model hosted on KServe, which identifies whether the traffic is benign or malicious. Based on this classification, KubeDeceive deploys appropriate decoys within the Kubernetes cluster. These decoys engage attackers, log their interactions, and protect real resources by creating an environment that feels authentic to adversaries. Feedback from these interactions is continuously analyzed to refine future deception strategies.

This seamless integration of detection and deception enables KubeDeceive to act as a proactive security layer, capable of adapting to changing attack patterns without manual intervention. The combination of real-time responsiveness and dynamic adaptability ensures the system remains effective even in high-traffic or rapidly evolving threat scenarios. By misleading attackers and capturing actionable intelligence, KubeDeceive not only mitigates immediate risks but also strengthens the framework’s overall security posture.

#### Intelligence gathering and threat analysis

KubeDeceive transforms interactions with decoys into actionable intelligence, strengthening the framework’s ability to adapt to evolving threats. By analyzing logs from attacker interactions, the system uncovers critical details about tools, techniques, and behaviors, enabling proactive adjustments to both detection and deception strategies.

These insights allow the system to refine decoy configurations to align with frequently targeted resources, such as Grafana or InfluxDB, ensuring decoys remain engaging and effective. Captured intelligence also enhances the detection pipeline by correlating interaction data with predictions from the KServe model, improving accuracy and responsiveness to emerging threats.

Real-time coordination is facilitated by a Go server integrated within the Webhook Pod, which manages mutation webhooks and orchestrates decoy deployments based on detection outcomes. This streamlined interaction ensures that the intelligence gathered is immediately actionable and contributes to both immediate defenses and long-term strategy refinement.

By leveraging these capabilities, KubeDeceive ensures Kubernetes environments are equipped to handle evolving threats with greater precision and adaptability.

### Integrated workflow

#### Workflow efficiency and resource scalability

The framework’s efficiency is achieved by breaking down its operation into four structured workflow phases: traffic capture, feature extraction, classification, and deception deployment. These phases work in unison to ensure seamless real-time threat detection and adaptive deception in Kubernetes environments.

**Fig. 4:**
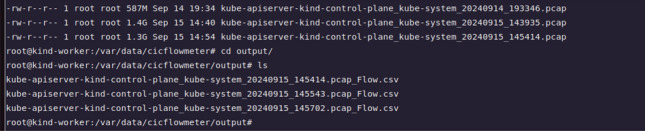
Conversion of PCAP Files to CSV using CICFlowMeter.

- **Traffic Capture:** Network traffic is captured directly from Kubernetes pods using KSniff, which intercepts packets from the cluster’s network interfaces. The captured traffic is stored as .pcap files in a Persistent Volume (PV) for subsequent analysis.
- **Feature Extraction:** The .pcap files are processed by CICFlowMeter as shown in Fig. 4, which converts raw packet data into structured NetFlow features. These features, including packet lengths, flow durations, and protocol types, are essential for identifying malicious patterns.
- **Classification:** Extracted features are passed through the detection pipeline, which includes feature scaling and dimensionality reduction using PCA and Autoencoders, followed by classification with a Naive Bayes classifier. This step determines the nature of the traffic-benign or one of several malicious categories-based on predefined CVEs.
- **Deception Deployment: **Based on classification results, the KubeDeceive module dynamically deploys decoys to mislead attackers and collect intelligence. These decoys mimic sensitive resources or services, such as vulnerable APIs or credentials, effectively engaging attackers while protecting real infrastructure.

To support this workflow, Kubernetes’ native features for resource scaling are leveraged, ensuring the system can handle varying workloads without compromising performance. Horizontal Pod Autoscaler (HPA) dynamically adjusts the number of active pods based on real-time resource usage metrics, such as CPU and memory consumption. Meanwhile, the Vertical Pod Autoscaler (VPA) optimizes resource allocations for individual pods, adapting to changing demands within the cluster. These auto-scaling features ensure the framework maintains low latency and high throughput, even during traffic spikes.

The framework’s adaptability is further enhanced by the MAPE-K loop. This loop continuously observes traffic patterns and attacker interactions, refining resource allocation and operational strategies. For example, during heightened reconnaissance activity, the loop triggers the deployment of additional decoys and scales detection resources to counteract the increased load dynamically.

By integrating an efficient workflow with Kubernetes’ advanced scaling features, the framework achieves both operational efficiency and adaptability. This ensures that the system remains robust and effective, providing real-time protection for Kubernetes environments against a diverse range of evolving threats.

#### Algorithmic steps and complexity analysis

The proposed framework for real-time traffic analysis and adaptive deception comprises four integrated phases: traffic capture, feature extraction, threat classification, and deception deployment. Each phase is discussed below, along with its computational complexity.

The process begins with traffic capture, where network packets from Kubernetes pods are collected using KSniff and stored as pcap files in a Persistent Volume (PV) for further processing. This step ensures that live traffic data is readily available for analysis. The time complexity of this phase is $O\left( n \right)$ , where $n$ represents the number of packets captured, and its space complexity scales with the size of the generated pcap files.

The second phase, feature extraction, converts raw pcap data into structured CSV features using CICFlowMeter. Extracted features include statistical metrics such as packet lengths, flow durations, and protocol types, which are crucial for detecting network anomalies. The time complexity is $O(m \cdot f)$ , where $m$ denotes the number of network flows and $f$ the number of features extracted. Space complexity corresponds to the size of the generated CSV files.

Next, in the threat classification phase, the extracted features are preprocessed by scaling and dimensionality reduction via PCA, followed by classification using a Naive Bayes model. The PCA operation has a time complexity of $O\left( n \cdot k^2\right)$ where $n$ is the number of samples and $k$ the number of PCA components. Naive Bayes classification operates with a time complexity of $O\left( n \cdot c \right)$ where $c$ represents the number of attack classes. The combined classification phase has a total complexity of $O\left( n \cdot \left( k^2 + c\right) \right)$ , with space complexity dependent on the size of the scaled and transformed feature set.

Finally, in deception deployment, KubeDeceive utilizes classification results to activate adaptive deception strategies. These strategies include deploying decoy services or rerouting malicious traffic. The dynamic adjustment of deception actions is guided by the MAPE-K loop (Monitor, Analyze, Plan, Execute, and Knowledge), which ensures continuous improvement. The complexity of this phase varies depending on the scale of the deployed decoy services and the computational overhead of monitoring and adjustments.

Overall, the framework efficiently integrates these phases, achieving a total time complexity of:

$$\begin{aligned} O(n + m \cdot f + n \cdot (k^2 + c)), \end{aligned}$$

where each term corresponds to traffic capture, feature extraction, and threat classification, respectively. The space complexity is determined by the cumulative storage of pcap files, extracted features, and resources allocated for decoys.

This workflow demonstrates the scalability and adaptability of the proposed framework in handling real-time traffic analysis and deception in Kubernetes environments.

## Framework implementation and methodology

In the Unified Multi-Class Detection and KubeDeceive Framework, the system is evaluated based on its ability to detect Kubernetes-specific attacks in real-time and respond with dynamic deception tactics. The KubeDeceive component focuses on intercepting Kubernetes-related actions, particularly those triggered via kubectl commands, rather than general HTTP-based reconnaissance. The experimental setup integrates KServe for multi-class detection, KSniff for traffic capture, and CICFlowMeter for feature extraction, ensuring comprehensive coverage of both detection and deception aspects in a Kubernetes environment. The methodology sections below describe how different deception actions are triggered and executed in response to these attack types:

### Detection and deception tactics

#### Detection and deception via kubectl commands

Deception tactics aimed at container escape and privilege escalation attempts, where attackers attempt to elevate privileges within the Kubernetes cluster or escape containerized environments. For privilege escalation attempts using kubectl commands like kubectl get secrets, kubectl create, or kubectl attach, KubeDeceive deploys decoy secrets or privileged pods. These decoys trick attackers into believing they have obtained sensitive access or privileges, but in reality, they are interacting with controlled, monitored environments. When a privileged command or secret access is detected, KubeDeceive redirects the attack to decoy secrets or privileged pods, isolating the attacker’s actions and logging their attempts to escalate privileges. The system is particularly effective against vulnerabilities like CVE-2019-11247 (Kubernetes privilege escalation)^24^ and CVE-2021-25741 (Kubelet privilege escalation vulnerability)^25^, where attackers attempt to gain unauthorized access to sensitive Kubernetes secrets or resources.

#### Detection and deception for network-based attacks related to CVEs

Deception actions based on network traffic analysis of specific CVEs. These attacks are detected using KSniff and CICFlowMeter, which capture network traffic and extract features for real-time analysis by the multi-class detection model hosted on KServe. The system analyzes captured traffic and identifies attack patterns related to specific CVEs, such as CVE-2020-13379 (Grafana SSRF)^2^, CVE-2021-43798 (Grafana path traversal)^3^ or CVE-2019-20933 (InfluxDB improper authentication)^26^. Based on these patterns, the system dynamically redirects traffic to decoy services that mimic the original ones. Attackers believe they are interacting with critical services but are instead engaging with decoy services that log their behavior. After traffic sniffing and feature extraction, if an attack related to a specific CVE is detected, the system redirects the attacker’s traffic to a decoy service. For instance, an SSRF attack targeting Grafana could be redirected to a decoy service that appears legitimate but has no real access to critical data.

### Experimental setup, metrics, and deception evaluation

This section outlines the experimental setup used to evaluate the Unified Multi-Class Detection and KubeDeceive Framework, focusing on metrics for effectiveness and deception tactics. The evaluation was conducted in a controlled Kubernetes environment, engaging security consultants with expertise in Kubernetes security and penetration testing. The consultants were tasked with executing various predefined attack scenarios. Unlike a traditional Capture the Flag (CTF)^27^ exercise, this evaluation focused on assessing the framework’s ability to detect and deceive across diverse attack vectors rather than achieving a single target.

**Environment Configuration:**

- Kubernetes Cluster: Configured with KServe for detection, KSniff for traffic capture, and CICFlowMeter for feature extraction.
- Vulnerable Pods: Deployed with misconfigurations (e.g., HostPID, HostNetwork). These decoys simulate vulnerable environments to engage attackers while protecting actual resources.
- Decoy Elements: Included fake secrets, misleading configurations, and dummy services to mimic critical applications like Grafana (CVE-2020-13379, CVE-2021-43798) and InfluxDB (CVE-2019-20933), redirecting malicious traffic and logging attacker actions.
- Audit Logging^28^: Enabled to capture detailed logs of interactions with decoy and real components.

**Evaluation metrics:**

1. Decoy-Specific Metrics

- Attacker Engagement Time: Average session durations, measured using decoy interaction logs.
- Decoy Success Rate: Calculated as the ratio of interactions redirected to decoys compared to real resources.
- Decoy Hit Rates: Computed by dividing the total interactions by the number of active decoys.
- Number of Active Decoys: Count of all decoys deployed during evaluation, tracked via deployment logs.
- Quality of Intelligence Gathered: A qualitative scale (1-5) assessing the relevance and utility of information (e.g., tools, behaviors).

2. Detection and Processing Metrics

- Detection Accuracy: Percentage of correctly identified malicious NetFlows.
- False Positive Rate: Proportion of benign NetFlows misclassified as malicious.
- Detection Latency: Time from pcap file processing to the classification of NetFlows.

3. System Resource Utilization Metrics

- CPU Usage vs. Pcap Size: Linear trend of CPU consumption with increasing pcap file sizes.
- Memory Usage vs. Pcap Size: Proportional increases in memory usage, validating resource efficiency under traffic loads.

This controlled setup allowed for a detailed analysis of the framework’s performance and provided insights into its ability to engage attackers effectively while safeguarding critical resources.

**Table 3:** Decoy performance metrics for selected attack scenarios.

Scenario	CVE ID	Engagement Time (seconds)	Decoy Success Rate (%)	Hit Rates (per decoy)	Intelligence Quality (1-5)
Server-Side Request Forgery (SSRF)	CVE-2020-13379	470	76	15	4.5
Path Traversal	CVE-2021-43798	360	75	17	4.4
Improper Authentication	CVE-2019-20933	275	78	18	4.6
Reconnaissance	N/A	495	80	20	4.7
Privilege Escalation via Kubernetes Secrets	CVE-2019-11247	310	90	18	4.8
Privilege Escalation via Kubelet	CVE-2021-25741	420	93	22	4.7

### Framework performance and resilience

The framework demonstrates robust performance and adaptability in Kubernetes environments, excelling in balancing detection accuracy, resource utilization, and latency. By leveraging Kubernetes-native features, such as Persistent Volumes (PVs) and Horizontal Pod Autoscaler (HPA)^29^, it efficiently manages high traffic volumes and dynamic workloads, ensuring real-time threat detection and adaptive deception.

The use of Persistent Volumes for traffic capture and feature extraction introduced several challenges that were addressed through targeted optimizations. Ensuring data consistency was critical to prevent overwrites during simultaneous access from multiple pods. This was mitigated by configuring appropriate access modes and managing file paths to prevent conflicts. Optimizing data flow was equally important to maintain low latency in the automated capture workflows, which was achieved by tuning storage backend settings and ensuring PVs were correctly sized and configured for expected loads. Furthermore, the system required scalable storage solutions to handle increasing volumes of captured traffic without disrupting ongoing processes, which was achieved by integrating Kubernetes-native scaling mechanisms.

The framework also faced potential vulnerabilities, including the risk of attackers recognizing decoys as fake and bypassing them. Randomizing decoy configurations and mimicking realistic service behaviors effectively mitigated this risk. Additionally, high traffic volumes posed a risk of overwhelming components like CICFlowMeter and PVs. Implementing load balancing and dynamic scaling for CICFlowMeter ensured uninterrupted operation during peak loads. The feedback-driven MAPE-K loop introduced another challenge, as attackers could potentially manipulate system responses. Introducing thresholds for anomaly classifications and cross-validating intelligence from multiple sources minimized this risk.

Through these mitigations, the framework ensures resilience and reliability, offering a scalable and secure solution for Kubernetes environments. Its ability to adapt dynamically while maintaining performance positions it as an effective defense mechanism for real-time threat detection and deception in modern cloud infrastructures.

## Evaluation

### Results and Key findings

The evaluation results varied based on sniffing durations, which ranged from 5 to 10 seconds. Shorter sniffing times provided faster processing but captured less comprehensive traffic data, leading to slightly reduced detection accuracy. Conversely, longer sniffing durations increased data richness, enhancing accuracy but introducing minor latency.

The framework was evaluated using three key metrics to assess its overall performance:

1. Decoy-Specific Metrics: These assessed the effectiveness of KubeDeceive, including attacker engagement time, decoy success rates, and the quality of intelligence gathered.
2. Detection and Processing Metrics: These measured the framework’s ability to classify traffic accurately and efficiently, focusing on detection accuracy, false positive rate, and latency.
3. System Resource Utilization Metrics: These evaluated the scalability and efficiency of the framework by analyzing CPU and memory usage under varying traffic loads.

1. **Decoy-Specific Metrics Results** Decoys are strategically deployed simulated resources designed to mimic real services or components within a Kubernetes environment. These decoys include fake secrets, privileged pods, misleading configurations, and dummy services, which aim to mislead attackers, prolong engagement, and gather actionable intelligence. By seamlessly integrating with the detection pipeline, decoys dynamically adapt to the nature of the threats detected, ensuring effective diversion of malicious actions.

The evaluation of decoy performance focused on metrics that measure the effectiveness of engagement and the quality of intelligence gathered during various attack scenarios. Table 3 provides a detailed breakdown of the results across multiple scenarios.

**Engagement time:** The system successfully extended attacker engagement with decoys. Average interaction times varied based on the attack type, with reconnaissance scenarios achieving the highest engagement time of 495 seconds, highlighting the decoys’ ability to sustain prolonged interaction.

**Success rate:** Decoy success rates were calculated as the proportion of attacker actions directed to decoys versus real resources. Rates ranged from 75% to an impressive 93%, depending on the scenario. Privilege escalation attempts via the Kubelet (CVE-2021-25741) demonstrated the highest success rate, indicating the effectiveness of dynamically deployed decoys tailored to specific threats.

**Table 4:** Performance metrics for selected attacks (15-second sniffing duration).

Scenario	CVE ID	Pcap Size (KB)	Detection Accuracy (%)	False Positive Rate (%)	Detection Latency (seconds)	CPU Usage (%)	Memory Usage (MB)
Server-Side Request Forgery (SSRF)	CVE-2020-13379	300	89	4.8	0.8	40	512
Path Traversal	CVE-2021-43798	320	90	5.2	0.9	45	640
Improper Authentication	CVE-2019-20933	280	87	5.0	0.8	39	480
Reconnaissance	N/A	650	91	6.5	1.2	55	896
Privilege Escalation via Kubernetes Secrets	CVE-2019-11247	400	90	6.0	1.1	50	768
Privilege Escalation via Kubelet	CVE-2021-25741	450	91	6.0	1.1	53	832

**Hit Rates per Decoy:** The average number of interactions per active decoy provided insight into the decoys’ ability to attract and engage attackers. The hit rates remained consistent across scenarios, with reconnaissance activity achieving the highest rate of 20 interactions per decoy.

**Intelligence Quality:** Decoys logged attacker behavior, tools, and techniques, which were assessed on a qualitative scale (1-5). Most scenarios scored between 4.4 and 4.8, demonstrating the decoys’ ability to collect high-value intelligence that informs future defensive strategies.

The metrics illustrate the robust functionality of the decoy system, with high engagement and success rates paired with valuable intelligence gathering. The results validate the framework’s ability to effectively deceive attackers and reinforce Kubernetes security.

2. **Detection and Processing Metrics Results**

The detection and processing metrics provide a detailed analysis of the framework’s ability to accurately identify malicious activity while maintaining efficiency in resource utilization. The results below demonstrate the system’s robustness across a variety of attack scenarios.

As shown in Table 4, detection accuracy consistently remained high, ranging from 88% to 91%. Web application attacks such as Server-Side Request Forgery (SSRF), path traversal and Improper Authentication, achieved accuracy (89%, 90% and 87%, respectively). Privilege Escalation via Kubelet (CVE-2021-25741), while more complex, demonstrated controlled detection with an accuracy of 91%. This was achieved by leveraging both kubectl command monitoring and traffic analysis, ensuring robust identification of privilege escalation attempts. Reconnaissance attacks, which often involve subtle and exploratory behaviors, showed a slightly higher accuracy of 89%, further highlighting the framework’s ability to adapt to diverse and evolving attack scenarios.

The false positive rate averaged 5.5%, highlighting the model’s reliability in distinguishing benign traffic from malicious activities. Simpler attack scenarios, such as SSRF, maintained a lower false positive rate of 4.8%, reflecting their distinct traffic patterns. In contrast, Reconnaissance showed the highest false positive rate at 6.5%, attributed to the overlap between exploratory behaviors and legitimate scanning activities, which is a common challenge in Kubernetes environments. This variance underscores the model’s strength in minimizing false positives while addressing the inherent complexity of detecting stealthier attack patterns.

Detection latency varied depending on the size of the pcap file and the complexity of the scenario. Smaller pcaps, such as those associated with SSRF, achieved a latency of 0.8s, supporting near real-time detection capabilities. In contrast, scenarios involving complex traffic patterns, like Privilege Escalation via Kubernetes Secrets (CVE-2019-11247), recorded a higher latency of up to 1.1s, indicating additional processing overhead for detailed analysis.

**Fig. 5:**
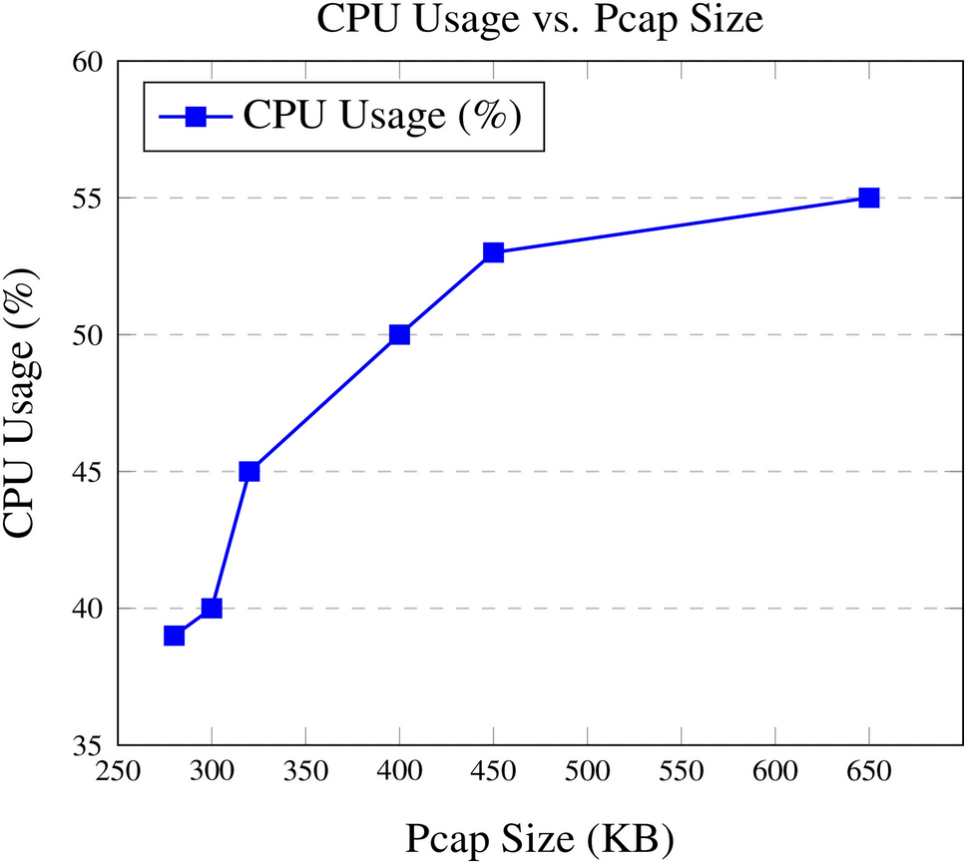
CPU usage vs. Pcap size with standardized axis ranges.

**Fig. 6:**
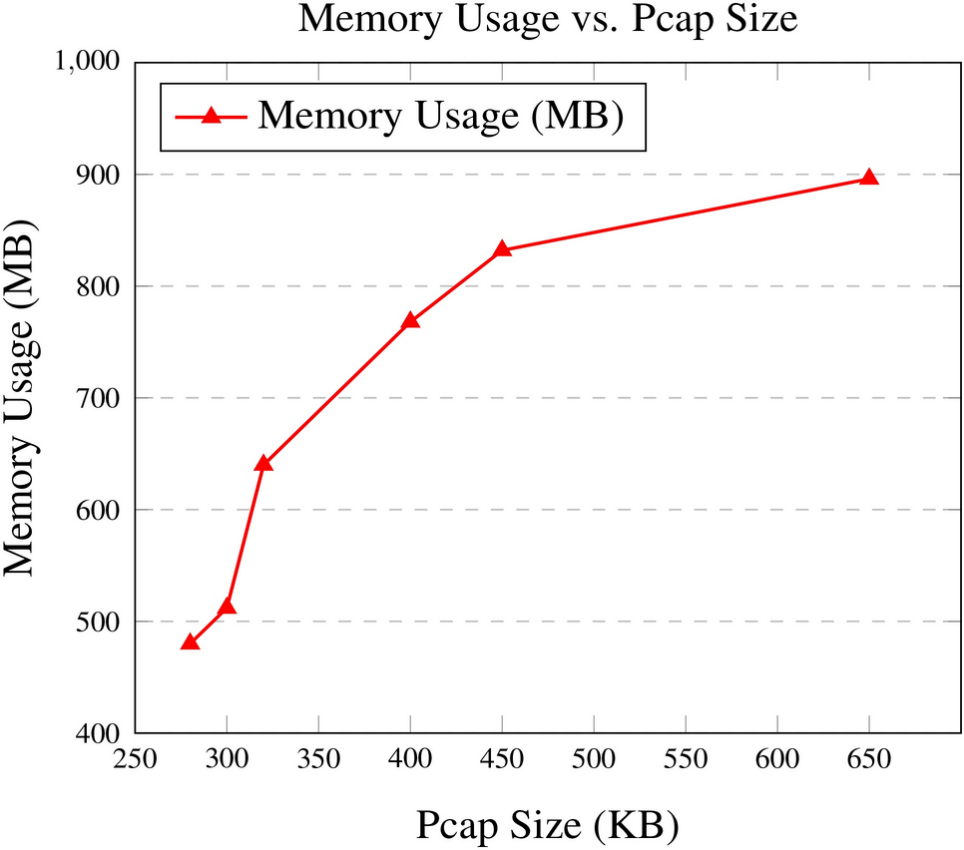
Memory usage vs. Pcap size with standardized axis ranges.

3. **System Resource Utilization Metrics Results**

The evaluation of system resource utilization demonstrates the framework’s ability to scale efficiently under varying traffic conditions. As pcap sizes increased, both CPU and memory usage showed predictable and proportional growth, ensuring stability even under high workloads.

As shown in Fig. 5, CPU Usage scaled linearly with traffic volume, ranging from 39% for smaller pcaps associated with simpler scenarios, such as improper Authentication (CCVE-2019-20933), to a peak of 55% during high-traffic scenarios like Reconnaissance. This linear growth confirms the system’s ability to handle larger data volumes without significant performance degradation.

Memory usage, illustrated in Fig. 6, was similarly proportional to pcap size, reaching a maximum of 896 MB for the most complex scenarios, such as Reconnaissance. Scenarios with lower traffic, such as Improper Authentication (CVE-2019-20933), exhibited minimal memory requirements, averaging around 480 MB. This efficient allocation of memory resources highlights the system’s practicality for deployment in resource-constrained environments.

Overall, the results validate the framework’s resource efficiency and scalability, ensuring that detection and deception mechanisms operate seamlessly without overburdening the Kubernetes infrastructure.

### Discussion of findings

The evaluation demonstrates that the proposed framework significantly enhances the security of Kubernetes environments by seamlessly integrating real-time multi-class detection and adaptive deception. Its ability to identify diverse attack scenarios, engage adversaries, and adapt dynamically sets it apart from existing solutions.

The detection model consistently achieved high accuracy across various attack types, ranging from 88% to 91%. This performance underscores the effectiveness of using a combination of Principal Component Analysis (PCA) and Naive Bayes classification for identifying malicious traffic patterns. Particularly, the framework excelled in handling simpler attacks, such as Server-Side Request Forgery (SSRF), while maintaining robust performance against more complex threats, including privilege escalation and reconnaissance.

The false positive rate, averaging 5.5%, demonstrates the reliability of the detection pipeline in distinguishing benign from malicious activities. This minimizes alert fatigue, a common challenge in security systems, thereby enhancing operational efficiency. The framework also maintained low detection latency, with response times as low as 0.8 seconds for smaller traffic captures, enabling near real-time threat response.

The integration of adaptive deception mechanisms further enhances the framework’s value. By deploying dynamic decoys tailored to specific threats, the system effectively diverts attackers, prolongs engagement, and gathers actionable intelligence. Metrics such as high decoy success rates (up to 91%) and attacker engagement times (up to 495 seconds) validate the impact of these mechanisms in mitigating immediate risks and supporting long-term defense strategies.

Resource utilization analysis highlights the scalability and efficiency of the framework, leveraging Kubernetes-native features such as Horizontal and Vertical Pod Autoscalers to dynamically adjust resource allocation based on workload demands. This ensures consistent performance even during high traffic loads, reinforcing the framework’s suitability for dynamic and resource-constrained cloud-native environments.

Overall, the proposed framework addresses critical gaps in Kubernetes security by unifying precise threat detection, proactive engagement, and scalable operations. This integrated approach not only mitigates known and emerging threats but also provides actionable insights that strengthen the security posture of Kubernetes clusters over time.

## Conclusion and future work

his study introduces an integrated framework tailored for Kubernetes environments, addressing critical challenges in dynamic and high-traffic cloud-native infrastructures. By unifying real-time threat detection with adaptive deception, the framework provides robust security against sophisticated attacks. The combination of KServe for detection, CICFlowMeter for feature extraction, and KubeDeceive for decoy deployment ensures precise threat identification and proactive engagement, with adaptability driven by the MAPE-K loop for continuous refinement.

The framework demonstrated significant performance in evaluations, achieving up to 91% detection accuracy across various attack scenarios, maintaining low false positive rates of 5.5%, and effectively redirecting attackers to decoys with success rates reaching 93%. The system also showed resource efficiency, with predictable scaling of CPU and memory usage and detection latency as low as 0.8 seconds. These results highlight the framework’s capability to deliver real-time protection while maintaining scalability and operational efficiency.

Future research will focus on extending the framework’s deception capabilities, automating model retraining, and integrating with broader security ecosystems such as SIEM systems^30^. These advancements aim to further enhance the framework’s effectiveness, ensuring its relevance in protecting Kubernetes environments against evolving threats.

## Data Availability

The datasets utilized during the current study are available in the GitHub repository, accessible at https://github.com/yigitsever/kubernetes-dataset.
